# Low genetic diversity contrasts with high phenotypic variability in heptaploid *Spartina densiflora* populations invading the Pacific coast of North America

**DOI:** 10.1002/ece3.4063

**Published:** 2018-04-20

**Authors:** Jesús M. Castillo, Blanca Gallego‐Tévar, Enrique Figueroa, Brenda J. Grewell, Dominique Vallet, Hélène Rousseau, Jean Keller, Oscar Lima, Stéphane Dréano, Armel Salmon, Malika Aïnouche

**Affiliations:** ^1^ Departamento de Biología Vegetal y Ecología Universidad de Sevilla Sevilla Spain; ^2^ Department of Plant Sciences MS‐4 USDA‐ARS Exotic & Invasive Weeds Research Unit University of California Davis California; ^3^ UMR CNRS 6553 ECOBIO Université Rennes 1 Rennes France; ^4^ Faculté de Médecine Institut de génétique et Développement de Rennes (IGDR) UMR6290, CNRS Université de Rennes1 Rennes France

**Keywords:** climate change, cordgrass, invasive species, microsatellites, phenotypic plasticity

## Abstract

Species can respond to environmental pressures through genetic and epigenetic changes and through phenotypic plasticity, but few studies have evaluated the relationships between genetic differentiation and phenotypic plasticity of plant species along changing environmental conditions throughout wide latitudinal ranges. We studied inter‐ and intrapopulation genetic diversity (using simple sequence repeats and chloroplast DNA sequencing) and inter‐ and intrapopulation phenotypic variability of 33 plant traits (using field and common‐garden measurements) for five populations of the invasive cordgrass *Spartina densiflora* Brongn. along the Pacific coast of North America from San Francisco Bay to Vancouver Island. Studied populations showed very low genetic diversity, high levels of phenotypic variability when growing in contrasted environments and high intrapopulation phenotypic variability for many plant traits. This intrapopulation phenotypic variability was especially high, irrespective of environmental conditions, for those traits showing also high phenotypic plasticity. Within‐population variation represented 84% of the total genetic variation coinciding with certain individual plants keeping consistent responses for three plant traits (chlorophyll *b* and carotenoid contents, and dead shoot biomass) in the field and in common‐garden conditions. These populations have most likely undergone genetic bottleneck since their introduction from South America; multiple introductions are unknown but possible as the population from Vancouver Island was the most recent and one of the most genetically diverse. *S. densiflora* appears as a species that would not be very affected itself by climate change and sea‐level rise as it can disperse, establish, and acclimate to contrasted environments along wide latitudinal ranges.

## 1 INTRODUCTION

Invasive plant species can respond to environmental pressures in novel environments through genetic change at individual level (local adaptation), gene expression regulation and epigenetic processes (Richards, 2012), and phenotypic plasticity in response to different environmental conditions (Fuller et al., 2010; Miner, Sultan, Morgan, Padilla, & Relyea, 2005; Stomp et al., 2008). In addition, novel genotypes resulting from hybridization between native and invasive plant species may facilitate rapid evolution (Strong & Ayres, 2013). These different evolutionary processes are closely related as phenotypic plasticity may itself evolve through genetic changes (Crispo, 2008) and may contribute to the evolution of traits that support increased niche breadth of invasive species as plasticity facilitates persistence of individuals facing novel environmental pressures (Drenovsky et al., 2012). Phenotypic plasticity is adaptive, affecting the performance and reproductive success of individuals, being a trait itself, and, therefore, is subjected to evolution (Matesanz, Gianoli, & Valladares, 2010). Additionally, intrapopulation trait variation may modulate the effects of genetic diversity on populations (Hughes, 2014). Moreover, polyploidization, and especially hybridization, have been related to high levels of genetic diversity, epigenetic variation, and phenotypic plasticity (Braeutigam et al., 2013; Meyerson et al., 2016) and they are considered important in shaping the geographic range of plant species (Weiss‐Schneeweiss, Emadzade, Jang, & Schneeweiss, 2013).

In this context, few studies have evaluated the relationships between genetic differentiation and phenotypic plasticity of invasive plant species to novel, changing environments along wide latitudinal ranges. These studies have shown that species with low genetic variation can colonize wide latitudinal ranges due to high plasticity levels, sometimes related to polyploidy and hybridization, and their spread into dissimilar environments can lead to genetic differentiation (Counts, 1993; De Kort et al., 2016; Meyerson et al., 2016; Quilot‐Turion et al., 2013; Zhao, Yang, Xi, Gao, & Sun, 2012). For example, the high level of epigenetic changes recorded in invasive *Spartina anglica* (Parisod et al., 2009; Salmon, Ainouche, & Wendel, 2005) has been related to its high levels of morphological plasticity and its large ecological amplitude (Thompson, McNeilly, & Gay, 1991), contrasting with low interindividual genetic diversity (Ainouche, Baumel, & Salmon, 2004; Ayres & Strong, 2001; Baumel, Ainouche, & Levasseur, 2001). In contrast, the cosmopolitan glycophyte *Phragmites australis* (Cav.) Trin. ex Steud. shows high genetic diversity, with some lineages behaving as invasive out of their native range, at the same time that their high phenotypic plasticity improves the responses to global change factors (Eller et al., 2017). Recently, Liu, Strong, Pennings, and Zhang (2017) have showed that both phenotypic plasticity and rapid local adaptation can contribute to the spread of an invasive species, being both processes interrelated and depending on environmental conditions changing with latitude.

The knowledge of these dialectic relationships between genetic and epigenetic changes and trait expression is especially important for making decisions about conservation of endangered species and for management of invasive species in a scenario of globally changing environment that is modifying the distribution of organisms (Kelly & Goulden, 2008). Thus, it is important to link functional trait‐based responses to genetic diversity and environmental changes to improve our understanding of mechanisms that promote changes in the species distribution and adaptation (Nicotra et al., 2010). For example, a better understanding of the role of phenotypic plasticity in response to environmental variation will improve our ability to manage weed invasions and improve conservation efforts. In fact, it has been hypothesized that global change and biological invasions are synergistic processes that interact and impart negative ecological and economic impacts (Vilà, Corbin, Ducks, Pino, & Smith, 2007). Thus, studies that integrate biological invasions, climate change, and phenotypic plasticity are needed (Engel, Tollrian, & Jeschke, 2011).

The invasion of the heptaploid austral cordgrass, *Spartina densiflora* Brongn. (syn. *Sporobolus densiflorus* following Peterson, Romaschenko, Arrieta, & Saarela, 2014), across a 12° wide latitudinal gradient along the Pacific coast of North America from San Francisco Bay (ca. 37°56′N, USA) to Vancouver Island (ca. 49°20′N, Canada) provides a natural model system for an integrated study of the potential mechanisms underlying the response of species traits to substantial variation in climate and other environmental variables and an opportunity to investigate how species establish and succeed in novel and contrasted environments. Previous studies in the field and in common‐garden experiments on invasive *S. densiflora* populations sampled along the Pacific coast of North America revealed high levels of phenotypic plasticity for some foliar and tussock traits (Castillo, Grewell, Pickart, Figueroa, & Sytsma, 2016; Castillo et al., 2014; Grewell, Castillo, Skaer‐Thomason, & Drenovsky, 2016). However, the levels of genetic diversity in these *S. densiflora* populations along the Pacific coast of North America experiencing high variability in sedimentary and climatic conditions have not been studied.

In this study, we performed genetic analyses of *S. densiflora* using chloroplast (cp) and nuclear DNA sequences, involving simple sequence repeats (SSRs). Cp‐DNA sequences (useful for identification of maternal lineages in a hybridization context and for phylogeographic analyses) and potentially highly polymorphic nuclear SSRs were employed to explore genetic diversity among populations established along the Pacific coast of North America. Phenotypic variation within and among populations of *S. densiflora* was also evaluated through analysis of 33 morphological, physiological, and biochemical traits, on plants sampled in the field that were compared to plants maintained in a common‐garden. Five invasive populations of *S. densiflora* along the Pacific coast of North America from San Francisco Bay to Vancouver Island were analyzed to examine the mechanisms allowing this species to deal with changing environmental conditions and the relationships between these mechanisms. Analyzing the level of intrapopulation phenotypic variability and the phenotypic variation of many plant traits between populations of the same species invading contrasted environments and in a common‐garden experiment, together with knowledge of genetic diversity, would allow us to know the nature of the above‐mentioned phenotypic variation and its level of phenotypic plasticity (Zhao et al., 2016). Combining these different levels of analysis (phenotypic and genetic, contrasted and common environments, and intra‐ and interpopulation) is particularly relevant and should shed light on the ways that a polyploid species with hybrid origin (intrinsic characteristics that can promote phenotypic variability at different levels; Grover et al., 2012) can respond to spatial and temporal environmental changes. The quantification of plasticity for several traits in several populations of the same species is a difficult task, and thus, available data in this regard are still very scarce (Molina‐Montenegro & Naya, 2012). Integrated biogeographic approaches involving the study of natural populations along latitudinal gradients paired with common‐garden experiments are a way to increase our understanding of plant species responses to environmental change, yet the spatial scale of most studies has been limited (Elsey‐Quirk, Seliskar, & Gallagher, 2011; Hierro, Maron, & Callaway, 2005).

Our overall objective was to assess whether trait responses were dependent on genetic differentiation or phenotypic plasticity or a combination of both factors. We hypothesized that (i) *S. densiflora* would show low genetic diversity and high phenotypic plasticity in response to contrasted environments and that they would be independent from each other, based on previous observations (Castillo et al., 2014, 2016; Grewell et al., 2016); (ii) due to the polyploid and hybrid nature of *S. densiflora*, at least certain foliar traits would show both high plasticity and high intrapopulation variability, as leaf (the organ of photosynthesis and transpiration) is highly sensitive to environmental conditions (Stephenson, Oliver, Burgos, & Gbur, 2006); and (iii) genetically based and environmental‐based interpopulation differences in response to a changing environment would increase with geographic distance.

## 2 METHODS

### 2.1 Studied sites

*Spartina densiflora* populations were sampled in summer of 2010 at five invaded salt marshes along the Pacific coast of North America, covering its entire latitudinal distribution in North America (along 12° of latitude). See Castillo et al. (2014, 2016) for a full description of sampling sites. The southernmost population analyzed was at Corte Madera Creek in San Francisco Bay Estuary (SF; 37°56′33″N, 122°30′55″W; California, USA) in a middle‐elevation salt marsh dominated by *S. densiflora*, which was intentionally introduced from Humboldt Bay to stabilize tidal creek banks in a wetland restoration project in the late 1970s (Faber, 2000). The next population to the north was located at Vance Marsh, Humboldt Bay (HB; 40°49′53″N, 124°10′17″W; California, USA) in a middle intertidal elevation marsh where the species has been probably introduced accidentally by merchant vessels from Chile during the late nineteenth century (Spicher & Josselyn, 1985). The third population was near the mouth of the Mad River Estuary (MR; 40°56′10″N, 124°7′48″W; California, USA) fringing the narrow intertidal zone at a river bank, just north of Humboldt Bay. We also included a population of a middle marsh elevation from Grays Harbor Estuary (GH, 46°57′N, 124°8′08″W; Washington, USA), where *S. densiflora* invasion was first recorded in 2001 (Pfauth, Sytsma, & Isaacson, 2007). Finally, the fifth and northernmost population was in a fringing salt marsh along the narrow Baynes Sound channel on the east coast of Vancouver Island (VI; 49°33″N, 124°52′09″W; British Columbia, Canada), region where *S. densiflora* was first discovered in 2005 (Morgan & Sytsma, 2010).

*Spartina densiflora* populations were visited during low tides. Ten adult tussocks with between 10 and 80 live shoots and a mean tussock area between 15 and 41 cm^2^ (ca. 2–3 years of age) were randomly selected, separated by a minimum of 1 m in attempt to sample potentially different genotypes, and individually marked in each of the five populations. Due to the low genetic diversity of *S. densiflora* populations recorded previously in San Francisco Bay and Humboldt Bay (Ayres et al., 2008), we assumed that *n* = 10 individuals would be representative of each population, a number of plants per population similar to that sampled in previous studies on genetic diversity of invasive species (Andreakis, Kooistra, & Procaccini, 2009; Guo et al., 2015; Moran, Reid, & Levine, 2017). The external limits of each *S. densiflora* tussock were clearly visible, so sampling tussocks separated by a minimum of 1 m ensured that plants coming from different establishment events were sampled.

### 2.2 Chloroplast DNA sequencing

DNA was extracted from dried leaves of the 10 marked individuals from each of the five populations by employing a NucleoSpin Plant Extraction Kit (Macherey‐Nagel GmbH & Co, Düren, Germany). Chloroplast DNA sequences were analyzed and compared with corresponding sequences from GenBank, obtained on *S. densiflora* samples from various regions (see below). The *trnT‐trnF* chloroplast DNA (Cp‐DNA) region was chosen as it is well documented in *Spartina* sequence databases (and then useful for chlorotype comparison between samples from different species and geographic locations). This region was amplified by polymerase chain reaction (PCR) in a Mastercycle thermal cycler (Eppendorf AG, Hamburg, Germany) and underwent denaturation for 2 min at 94°C, followed by 35 cycles at 94°C for 30 s, 54°C for 30 s, and 72°C for 1 min 30 s, followed by an extension phase at 72°C for 7 min. PCR products were purified using a NucleoSpin Gel and PCR purification kit (Macherey‐Nagel GmbH & Co) and sequenced directly (on both directions) by Sanger technique at Macrogen Europe sequencer (Amsterdam, The Netherlands). The amplification of the *trnT‐F* region was performed in two sections using the primer pairs a and b to recover the *trnT‐trnL* segment and primer pairs *c* and *f* to recover the *trnL‐trnF* segment (Taberlet, Gielly, Pautou, & Bouvet, 1991). Sequence alignments and comparisons were performed with the Geneious 9.1.2 software (Kearse et al., 2012). Sequences were compared with previously published *S. densiflora* sequences (Ayres et al., 2008;, Baumel et al., 2001; Fortune et al., 2008).

### 2.3 Simple sequence repeats

Neutral molecular markers such as SSRs provide independent and complementary assessment of genetic diversity to phenotypic variation most likely submitted to selective constraints. They are thus commonly used for genotyping in studies of phenotypic variations and fitness (Agarwal, Shrivastava, & Padh, 2008; Chapman, Nakagawa, Coltman, Slate, & Sheldon, 2009). A total of 1435 microsatellite repeats (SSRs) of 4–6 bp were identified from *Spartina maritima* genomic data obtained by Illumina sequence assemblies (A. Salmon, *unpublished*), using the MISA (MIcroSAtellite Identification Tool) software (Thiel, Michalek, Varshney, & Graner, 2003). Primer pairs were designed using the default parameters (and optimized TM ranging between 55 and 60°C) of the PRIMER3 software (Koressaar & Remm, 2007; Untergasser et al., 2012), which resulted in a range of primer product sizes of 100–280 bp and SSR motifs of at least −4 to 6 nt long, repeated five to eight times. This resulted in 395 different microsatellite loci with successful primer design (A. Salmon, *unpublished data*). For the first test, we selected 20 primer pairs considering those with the highest number of expected amplicon size and number of motif repetitions. PCR was performed as described above with these 20 primer pairs in *S. densiflora* nuclear DNA and analyzed by electrophoresis in agarose gel (1%, 35 min, 100V). Eight primer pairs of which amplification products produced unique and clear bands were chosen for further analysis (Table 1).

**Table 1:** Nucleotide sequences of the selected primer pairs and their corresponding microsatellite repeated motif for the 8 loci analyzed in five invasive populations of *Spartina densiflora* along the Pacific coast of North America

Locus	Forward primer sequence	Reverse primer sequence	SSR
MS2	ATATTCCGATCCCCTCCTTG	TTCGATCGGTCATGTTTTGA	(AAAG)_n_
MS7	CAGAATCACCATCATCAGCG	TTCCATTTTTCAGGGTGAGC	(TGGCAG)_n_
MS13	CTTGACCGCAACCAGTATGA	CCCAGGGCAATGGTTATACA	(TTCT)_n_
MS14	TGAGTTTGAGTTCACGGTTCA	ATGTGATGCCATTTCCACAA	(AAAG)_n_
MS15	TGCATTGCAGCAAGAGAATC	CGCTAGCTGATCCTGGAAAC	(GATG)_n_
MS16	GGGACACGGGATAGGAAAGT	CCGCCGTGCAATTATTTATC	(GTGGA)_n_
MS17	TTTGTTCAGCTTCAGCATGG	TTCTTGCAGTCGTTCTGTGC	(GAAA)_n_
MS18	TCTTATGGACCCCTTGCAGT	CATCCGATTGGCGTAAGATT	(TGATA)_n_

The 50 *S. densiflora* DNA samples previously extracted were diluted to a concentration of 10 ng/μl and amplified using the eight microsatellite primer pairs selected. The protocol used for microsatellite locus amplification by Baumel et al. (2016) was modified as follows: Amplifications were performed in 15 μl that contained 0.4 ng/μl DNA, 1× GoTaq Flexi Buffer (Promega), 0.02 U/μl GoTaq (Flexi, Promega), 0.2 mmol/L dNTPs, 2 mmol/L MgCl_2_, and 0.04 μmol/L reverse and forward primers. FAM‐labeled reverse primers were used. Amplifications were conducted in a Mastercycle Eppendorf thermal cycler, using the following program: 94°C 2 min., followed by 35 cycles 94°C 30 s, 50°C 30 s, and 72°C 1.30 min.

The amplified products were diluted to 1/30 before subsequent analysis. An aliquot of 2 μl of these diluted PCR products was mixed with 10 μl of formamide solution (975 μl formamide + 25 μl of GeneScan‐500 LIZ size marker) and separated by electrophoresis in an ABI PRISM 16‐capillary 3130xl Genetic Analyzer (Applied Biosystems Inc., Waltham, USA). Alleles were identified and binned using GeneMapper 4.1 software (Applied Biosystems Inc., Waltham, USA).

### 2.4 Genetic diversity analyses

SSR alleles were determined by comparison with the standard marker size (GeneScan‐500 LIZ size standard), and the different “genotypes” (harboring different allele combinations) were scored. *S. densiflora* is a heptaploid species (2*n* = 7*x* = 70), so up to seven alleles may be expected per locus. As allelic dosage could not be ascertained from the obtained chromatograms, SSRs were treated as “dominant markers” (i.e., alleles were scored as either present or absent) using the GenAlEx 6.502 software (Peakall & Smouse, 2006, 2012). Genetic diversity analyses are less affected by polyploidy using this approach (Obbard, Harris, & Pannell, 2006).

The intrapopulation genetic diversity parameters, effective number of alleles, Shannon Information Diversity Index (Brown & Weir, 1983), expected heterozygosity (Hartl & Clark, 1997), and unbiased expected heterozygosity (Peakall & Smouse, 2012) were obtained for each population.

A cluster analysis was performed using the *phangorn* R package (Schliep, 2011) with the matrix of pairwise genetic differences and the unweighted pair‐group method with arithmetic averages (UPGMA; Sneath & Sokal, 1973). The proportion of genetic diversity among and within populations was assessed by molecular variance analysis (AMOVA) using the GenAlEx 6.502 software for the five sampled populations of *S. densiflora*. Genetic differentiation among populations (Φ_PT_), analogous to Wright's *F*’_ST_ for dominant data, was obtained following standardization (Meirmans, 2006).

### 2.5 Phenotypic variation analyses

The following 33 tussock and foliar phenotypic traits were evaluated in the field for the genetically analyzed individuals of *S. densiflora*: shoot density in (1) alive, (2) spiked, and (3) dead stem density (shoot per m^2^), shoot (4) length (cm) and (5) diameter (mm), number of (6) live and (7) dead leaves, (8) total nonstructural carbohydrates in rhizomes (mg/g), (9) root biomass (g/m^2^), (10) rhizome biomass (g/m^2^), (11) belowground biomass (BGB, g/m^2^), (12) dead shoot mass (g/m^2^), (13) live shoot biomass (g/m^2^), (14) dead leaf mass (g/m^2^), (15) live leaf biomass (g/m^2^), (16) aboveground biomass (AGB, g/m^2^), (17) AGB: BGB ratio, (18) leaf area index (LAI) (m^2^/m^2^), (19) maximum leaf wide (cm), (20) leaf length (cm), (21) leaf area (cm^2^), (22) specific leaf area (SLA) (m^2^/g), (23) leaf longitudinal adaxial rolling (%), (24) leaf elongation rate (mm/day), (25) leaf water content (LWC) (%), content of (26) chlorophyll (Chl) *a* (mg/g DW), (27) Chl *b* (mg/g DW), (28) Chl *a*+*b* (mg/g DW), and (29) carotenoids (Car) (mg/g DW), (30) Chl:Car ratio, and (31) Chl *a*: Chl *b* ratio, and foliar content of (32) carbon and (33) nitrogen (mg/g). Leaves traits were measured in flag leaves to minimize differences due to the foliar ontogeny. These plant functional traits were chosen as they together describe appropriately the leaf characteristics and the tussock architecture of *S. densiflora*, being key factors in shaping the responses of this clonal species to its surrounding environment. Detailed methods and values of these plant traits for the studied populations of *S. densiflora* were reported previously by Castillo et al. (2014, 2016) and they are analyzed in this study using phenotypic plasticity indices and in relation to genetic analyses.

A common‐garden experiment was carried out to analyze whether the variation in the plant traits of the five studied populations of *S. densiflora* was the same at contrasted environmental conditions in the field than in a common environment. Thus, the common‐garden experiment allowed us to study the nature of the changing responses of *S. densiflora*, analyzing whether its variations in different plant traits were fixed independently of the surrounding conditions or whether they depended on the environment. With this aim, every tussock of *S. densiflora* measured in the field was collected and rhizomes were separated and grown in perlite substrate for 27 months (plastic pots: 20 cm diameter × 18 cm height) in a common‐garden experiment in the glasshouse facility of the University of Seville, Spain (37º21′42′′N, 5º59′15′′W). Pot bases were kept permanently flooded to a height of 2 cm and watered with 20% strength modified Hoagland's nutrient solution (Epstein, 1972; Hoagland & Arnon, 1950). Solutions were changed once a week. After 27 months of growth, every tussock trait recorded in the field, except belowground biomass, rhizome TNC, and leaf carbon and nitrogen concentration, was recorded again for the same plants acclimated to common glasshouse conditions (*n* = 10 tussocks per population). Common glasshouse conditions included substrate redox potential of 234 ± 5 mV, pH 8.3 ± 0.2, electrical conductivity 0.5 ± 0.0 mS/cm, mean monthly air temperature 23 ± 2°C, and mean monthly air relative humidity 62 ± 2%. Light conditions of mean radiation of 700 μmol photon m^−2^ s^−1^ at canopy level and a daily photoperiod of 16 hr (that was extended with incandescent lights, Osram Vialox NAV‐T (SON‐T) 400 W, giving a continuum spectrum) were set up to imitate those recorded at higher latitude sites during summer in relation to photoperiod and light intensity. *Spartina densiflora* is a facultative halophyte that can germinate, establish, and develop in freshwater conditions (Castillo, Rubio‐Casal et al., 2005; Nieva, Castellanos, & Figueroa, 2001). Freshwater was used to avoid salinity effects on tussock and foliar growth.

Phenotypic distances between plants in the field were calculated as Gower similarity index ranging between 0 and 1 (Gower, 1971) using the package *vegan* (Oksanen, 2007) of R software to obtain a pairwise dissimilarity matrix.

Correlations between genetic, phenotypic, and geographic distances in the field were performed using the Pearson's correlation coefficient (*p* < .05). Comparisons of the dissimilarity matrices described above were tested by Mantel's test for matrix correspondence (Mantel, 1967) using GenAlEx 6.502 software with 1000 permutations, following the method of Smouse and Long (1992); Smouse, Long, and Sokal (1986). A cluster analysis was performed using the package *vegan* (Oksanen, 2007) of the R software with the matrix of phenotypic pairwise dissimilarity.

Interpopulation phenotypic variability index (or phenotypic plasticity index *sensu* Valladares, Sanchez‐Gomez, & Zavala, 2006) (PI_*v*_) was calculated for each plant trait to evaluate differences between populations in phenotypic trait expression in the field and in the common‐garden experiment. PI_*v*_ for each trait was quantified as the percentage of change in absolute high and low mean trait values (Molina‐Montenegro & Naya, 2012):

$${\text{PI}}_{v}=\left[\left(X_{\max}-X_{\min}\right)/X_{\max}\right]\times 100$$

where *X*_max_ is the maximum mean value for a given trait and *X*_min_ is the minimum mean value for a given trait at population level (Valladares et al., 2006). This index is adequate for comparison of plasticity *sensu lato*, which is more focused on the responses of species to variable environments and allows traits with divergent ranges in variation and/or that are expressed in different units to be compared (Valladares, Dobarro, Sanchez‐Gomez, & Pearcy, 2005; Valladares et al., 2006). The PI_*v*_ recorded in the common‐garden experiment was not the product of phenotypic plasticity in response to different environments, but it was calculated as a way to measure to what extent the PI_*v*_ recorded in the field was related to phenotypic plasticity or with interpopulation differences in intrapopulation phenotypic variability.

Intrapopulation phenotypic variability index (IVI) was calculated also for each plant trait and population in the field and in the common‐garden experiment as the percentage of change in absolute high and low trait values:

$$\text{IVI}=[\left(x_{\max}-x_{\min}\right)/x_{\max}]\times 100$$

where *x*_max_ is the highest value for a given trait and population and *x*_min_ is the lowest value for a given trait and population. To estimate the overall intrapopulation variability for each trait in the field and in the common‐garden experiment, we calculated the arithmetic average of the intrapopulation variability observed for all five populations. To estimate the intrapopulation variability for each population, we calculated the arithmetic average of the values recorded for all 26 or 33 traits (in the common‐garden experiment and in field, respectively). The overall mean intrapopulation variability in field and in the common‐garden experiment was calculated as the arithmetic average of the variability for every plant trait.

Intrapopulation variability was compared among traits and among populations using one‐way analysis of variance (ANOVA) (*F*‐test, *p* < .05) using the plant trait or the geographic location as the grouping factor, respectively. Dependent variables were tested for homogeneity of variance using Levene's test and for normality using Kolmogorov–Smirnoff test. The relationships between PI_*v*_ and intrapopulation variability index in the field and in the common‐garden experiment were analyzed using Pearson's correlation coefficient (*r*) and linear regressions. Total intrapopulation variability and total phenotypic plasticity (calculated as arithmetic means for all traits and populations together) were compared using paired‐samples *t*‐test. Deviations were calculated as standard error of the mean (*SE*). The α‐level of significance was *p* < .05 for all tests. These analyses were carried out using SPSS 12.0 (SPSS Inc., Chicago, USA).

## 3 RESULTS

### 3.1 Genetic diversity

#### 3.1.1 DNA sequencing

The chloroplast DNA sequences analyzed for the *trnT‐L* (GenBank accessions MG869717 to MG869721) and *trnL‐F* (GB accessions MG869712 to MG869716) regions were identical in the five populations examined in this study. No difference was encountered between these sequences and those previously reported for *S. densiflora* samples from California (Humboldt Bay, Baumel et al., 2001; San Francisco, Ayres et al., 2008) and Argentina–Chile (Fortune et al., 2008).

#### 3.1.2 Simple sequence repeats

The eight microsatellites primer pairs yielded a total of 19 different alleles. Each primer pair amplified between one and five different alleles per locus, with an average of two alleles per locus. The eight loci examined exhibited different levels of genotype diversity: One single genotype was detected for all populations with the markers MS2, MS7, MS16, and MS17, two different genotypes at the MS13, MS14, and MS18 loci and three genotypes were encountered for MS15 (Table 2). We found seven different multilocus genotypes when all loci recorded were considered, with the most common multilocus genotype (I) present in 40% of the examined individuals (Table 3). The population from Grays Harbor presented two specific multilocus genotypes (II and VI) that were not present in any other population (Table 3).

**Table 2:** Genotype frequencies at eight microsatellite loci in five populations of *Spartina densiflora* along the Pacific coast of North America

	Genotypes (fragment sizes in bp)	VI	GH	MR	HB	SF
MS2	186	1	1	1	1	1
MS7	248/261	1	1	1	1	1
MS13	114/245	0.5	0.4	0.3	0	0.5
	245	0.5	0.6	0.7	1	0.5
MS14	263/270	0.1	0	0.1	0	0.4
	270	0.9	1	0.9	1	0.6
MS15	137/140/223/230/253	0.1	0	0	0.14	0.11
	223/230/253	0.8	1	0.89	0.72	0.78
	223/230/239/253	0.1	0	0.11	0.14	0.11
MS16	240/249	1	1	1	1	1
MS17	275	1	1	1	1	1
MS18	218/254	1	0.25	1	1	1
	218/254/275	0	0.75	0	0	0

Populations: VI, Vancouver Island; GH, Grays Harbor Estuary; MR, Mad River Estuary; HB, Humboldt Bay Estuary; SF, San Francisco Bay Estuary

**Table 3:** Multilocus genotypes and their proportion for five populations of *Spartina densiflora* introduced along the Pacific coast of North America

Genotype	Multilocus genotype	VI	GH	HB	MR	SF	Total
I	0101001101111110111	0.33	0.13	0.43	0.78	0.29	0.400
II	0101001101111111111	0.00	0.50	0.00	0.00	0.00	0.100
III	0101001111111110111	0.00	0.00	0.14	0.11	0.00	0.050
IV	0101111101111110111	0.11	0.00	0.00	0.00	0.14	0.075
V	1101001101111110111	0.45	0.12	0.43	0.00	0.28	0.225
VI	1101001101111111111	0.00	0.25	0.00	0.00	0.00	0.050
VII	1111001101111110111	0.11	0.00	0.00	0.11	0.29	0.100

Alleles were scored as present = 1 or absent = 0 for the eight microsatellite loci.

Populations: VI, Vancouver Island; GH, Grays Harbor Estuary; MR, Mad River Estuary; HB, Humboldt Bay Estuary; SF, San Francisco Bay Estuary.

**Figure 1:**
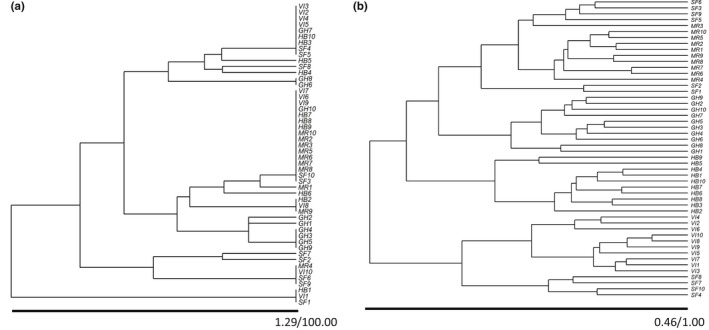
Cluster analysis (UPGM) of *Spartina densiflora* individuals based on (a) Huff genetic distance calculated on eight SSRs loci and (b) on Gower dissimilarity index based on 32 phenotypic traits for 50 individuals from five populations recorded in the field along the Pacific coast of North America. Populations: VI, Vancouver Island; GH, Grays Harbor Estuary; MR, Mad River Estuary; HB, Humboldt Bay Estuary; SF, San Francisco Bay Estuary

The population from Mad River had the lowest genetic diversity (Table 4), showing only three multilocus genotypes (Table 3) with seven indistinguishable plants following our molecular markers, nine of 10 plants grouped together in the same cluster and with individuals present only in two clusters in the UPGMA analysis (Figure 1). The other four populations (SF, HB, GH, and VI) presented higher intrapopulation genetic diversity (Table 4), all of them showing four multilocus genotypes, except HB with three genotypes (Table 3) and their individual plants scattered in many different clusters (Figure 1). The population from Vancouver Island presented one group of four plants and another group of three plants sharing the same genetic markers. The population from Grays Harbor showed two groups with four and two plants with the same markers, the population from Humboldt Bay had also two groups with three and two plants with identical markers, and the six of the 10 plants from San Francisco Bay were grouped in three pairs sharing the same genetic profile.

**Table 4:** Number of different alleles (N), number of effective alleles (Na), Shannon Information Diversity Index (I), expected heterozygosity (He), unbiased expected heterozygosity (uHe) (values are mean ± *SE*), and pairwise matrix of Nei unbiased genetic distances between five populations of *Spartina densiflora* introduced along the Pacific coast of North America

	VI	GH	HB	MR	SF
N	9.842 ± 0.086	9.368 ± 0.175	8.579 ± 0.279	9.684 ± 0.110	9.579 ± 0.116
Na	1.211 ± 0.123	0.895 ± 0.130	1.105 ± 0.130	1.000 ± 0.132	1.211 ± 0.123
Ne	1.060 ± 0.037	1.072 ± 0.055	1.053 ± 0.030	1.018 ± 0.010	1.085 ± 0.045
I	0.074 ± 0.035	0.060 ± 0.042	0.070 ± 0.034	0.033 ± 0.018	0.095 ± 0.043
He	0.042 ± 0.023	0.041 ± 0.029	0.040 ± 0.021	0.016 ± 0.009	0.057 ± 0.028
uHe	0.045 ± 0.024	0.043 ± 0.031	0.043 ± 0.022	0.017 ± 0.009	0.060 ± 0.029
VI	–				
GH	0.013	–			
HB	0.000	0.012	–		
MR	0.002	0.013	0.001	–	
SF	0.000	0.015	0.000	0.003	–

Populations: VI, Vancouver Island; GH, Grays Harbor Estuary; HB, Humboldt Bay Estuary; MR, Mad River Estuary; SF, San Francisco Bay Estuary.

**Figure 2:**
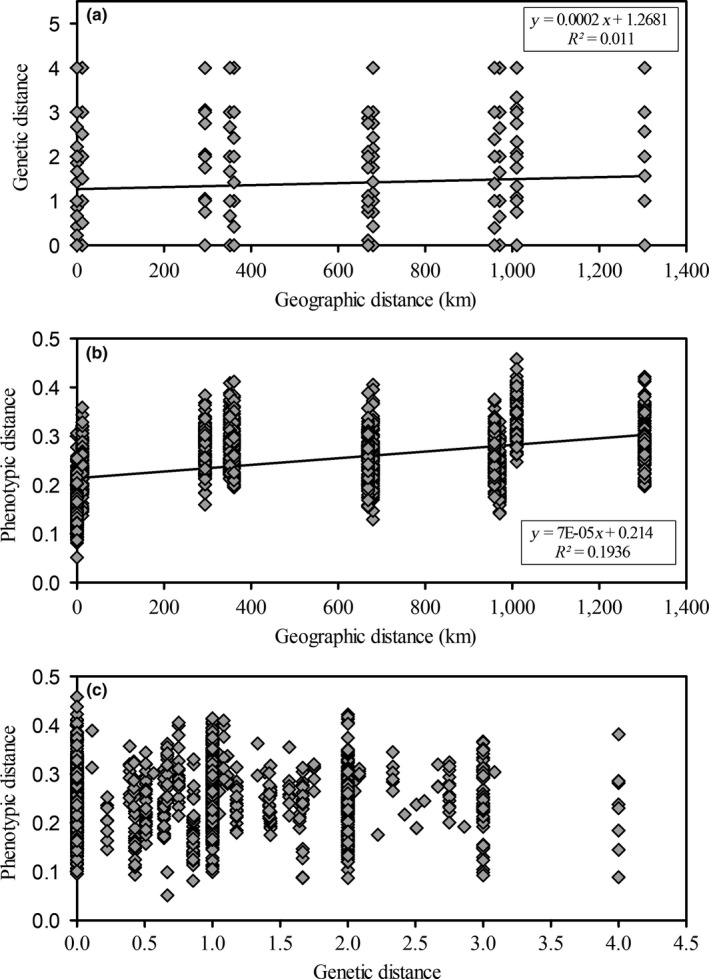
Mantel test for similarity between (a) genetic and geographic distances (*p* = .030), (b) phenotypic and geographic distances (*p* = .010), and (c) phenotypic and genetic distances (*p* = .403) for 50 individuals from five populations of *Spartina densiflora* recorded in the field along the Pacific coast of North America (*n* = 50)

Some genetic differentiation was observed among populations (Φ_PT_ = 0.159), but the AMOVA showed that interpopulation variance contributed to only 16% of the total genetic variation, whereas within‐population variation represented 84% of the total variation (Table 5). In fact, the five populations of *S. densiflora* were genetically very similar as showed by Nei's standard genetic distance (from 0 between SF and HB, SF and VI, and HB and VI to 0.15 between SF and GH; Table 4). The UPGMA analysis based on genetic distances revealed that each cluster was composed of individuals from different populations, which agreed with low interpopulation genetic differentiation (Figure 1). The Mantel test found a significant relationship between genetic distance and geographic distance (*p* = .030, *n* = 50; Figure 2a).

**Table 5:** Analysis of molecular variance (AMOVA) illustrating the proportion of genetic variation distributed within and among populations of *Spartina densiflora* along the Pacific coast of North America

Source	*df*	SS	MS	Est. Var.	Percentage
Among populations	4	6.943	1.736	0.113	16
Within populations	45	27.086	0.602s	0.602	84
Total	49	34.029		0.715	100

### 3.2 Phenotypic variation

Contrasting to genetic variation, consistent phenotypic differentiation among populations was encountered in the field. The dendrogram using phenotypic distances clustered together every individual of the same population, except for the population from San Francisco Bay of which individuals were grouped in different two clusters with plants from Mad River and Vancouver Island (Figure 1b). The Mantel test showed that phenotypic distance increased with geographic distance (*p* = .010, *n* = 50; Figure 2b), whereas no significant relationship was found between genetic distance and phenotypic distance in the field (*p* = .403, *n* = 50; Figure 2c).

**Figure 3:**
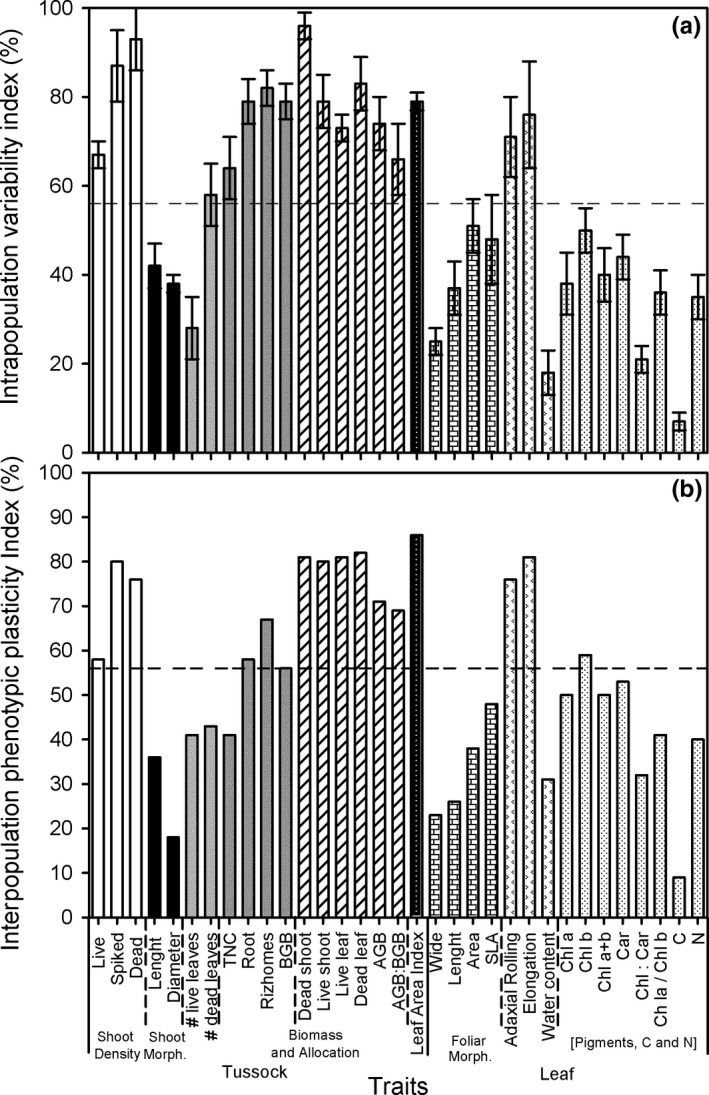
(a) Mean intrapopulation variation index (%) (±*SE*,* n* = 5) and (b) interpopulation phenotypic plasticity index (%) for 33 tussock and leaf traits from five *Spartina densiflora* populations recorded in the field along the Pacific coast of North America, organized into trait response groups. The horizontal dashed lines indicate the mean value for every trait

Mean IVI for the 33 recorded plant traits ranged from 7 ± 2% for foliar carbon content in the field to 96 ± 3% for dead shoot mass in the field (Figure 3a). PI_v_ in the field was from 9% for foliar carbon content to 86% for the leaf area index. In general, the traits with higher PI_*v*_ were those related to tussock aboveground architecture (shoot densities, above‐ and belowground biomass, and the leaf area index), and leaf adaxial rolling and elongation. In contrast, those traits showing lower PI_*v*_ in the field were related to shoot and leaf morphologies, leaf number per shoot, leaf water content, Chl:Car ratio, and foliar carbon content (Figure 3b).

**Figure 4:**
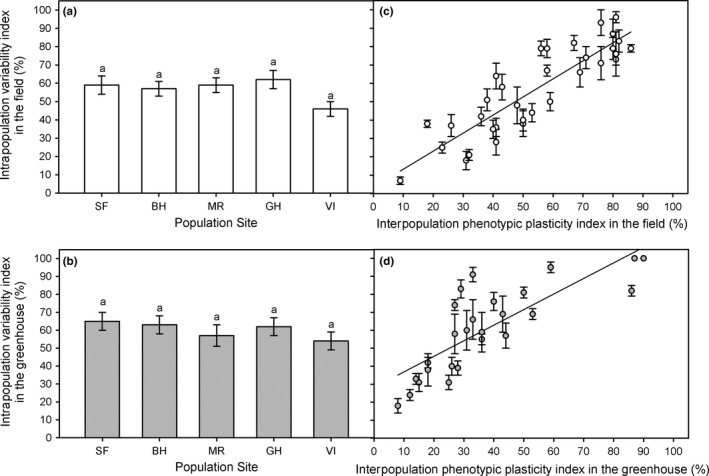
Mean intrapopulation variation index (%) (±*SE*,* n* = 5) for five *Spartina densiflora* populations recorded in the field along the Pacific coast of North America (a) and in a common‐garden experiment (b) calculated using their minimum and maximum values from their 33 plant traits (±*SE*,* n* = 33). The same letter above the bars denotes nonsignificant differences among populations (ANOVA, *p* > .05). Source populations are as follows: San Francisco Estuary, California (SF); Humboldt Bay, California (HB); Mad River Estuary, California (MR); Grays Harbor, Washington (GH); and Vancouver Island, British Columbia (VI). Relationship between mean intrapopulation variation index (%) and interpopulation phenotypic plasticity index (%) for 33 traits from five *Spartina densiflora* populations recorded in the field along the Pacific coast of North America (c) and in a glasshouse common‐garden experiment (d). Regression equation: (c) *y* = 3.38 + 0.98*x*; (d) *y* = 28.16 + 0.87*x*

Every population showed similar mean IVI from trait expression in the field (*F* = 1.644, *df* = 4, *p* = .166) and in the common‐garden experiment (*F* = 0.711, *df* = 4, *p* = .586), with similar mean values for the five studied populations in the field (56 ± 3%) and in the common‐garden experiment (60 ± 2%) (*t*‐test, *p* > .05) (Figure 4a,b). Mean IVI for plant traits was positively correlated with PI_v_ in the field (*r* = .878, *n* = 33, *p* < .0001) (Figure 4c) and in the common‐garden experiment (*r* = .804, *n* = 26, *p* < .0001) (Figure 4d). The IVI showed similar values than PI_*v*_ for every plant trait in the field (Figure 4c).

**Figure 5:**
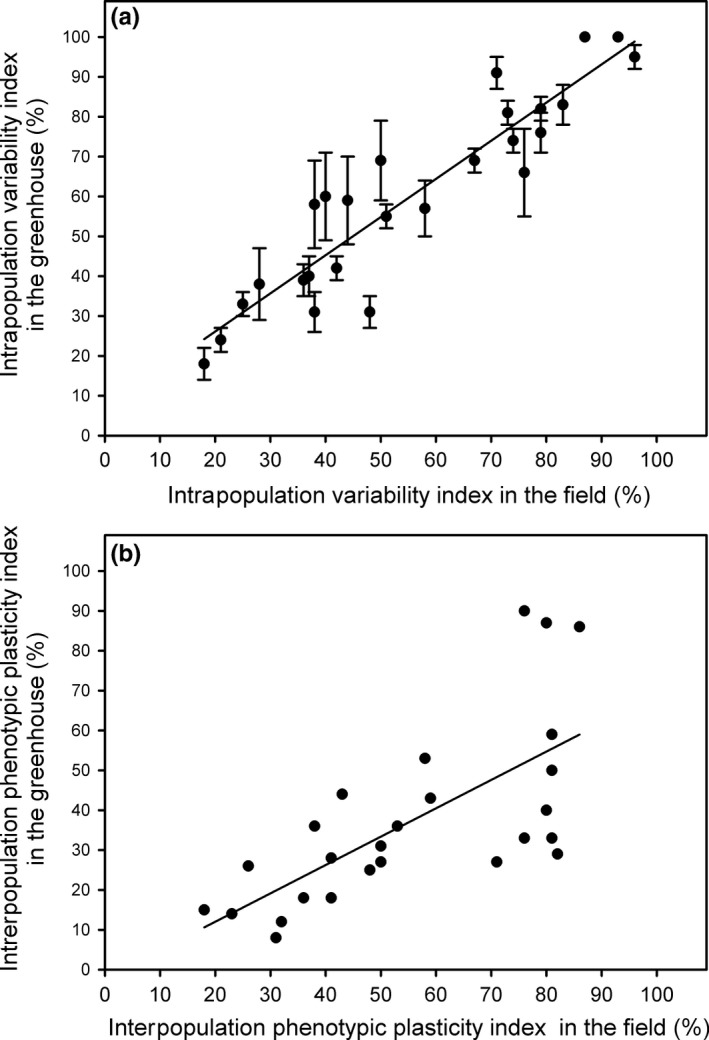
Relationships between intrapopulation variability index (a) and interpopulation plasticity index (b) in a common‐garden experiment and in the field for 26 traits from five *Spartina densiflora* populations along the Pacific coast of North America. Regression equation: (a) *y* = 6.98 + 0.96*x*; (b) *y* = −2.21 + 0.71*x*

On the other hand, those plant traits that had higher IVI and higher PI_*v*_ in the common‐garden experiment also had higher values for both variables in the field (*r* = .923, *p* < .0001, *n* = 26; *r* = .688, *n* = 26, *p* < .0001, respectively; Figure 5a,b). In addition, the IVI had similar values for every plant trait in the field and in the common‐garden experiment (Figure 5a), whereas PI_*v*_ tended to be lower in the common‐garden experiment than in the field (Figure 5b). Thus, total intrapopulation variability and total phenotypic plasticity indices for every plant trait and every population were similar in the field (56 ± 4% and 54 ± 4%, respectively; *t*‐test, *p* > .05). In contrast, total intrapopulation variability was higher than total interpopulation variability in the common‐garden experiment (60 ± 2% and 37 ± 4%, respectively; *t*‐test, *t* = 8.036, *df* = 25, *p* < .0001).

**Figure 6:**
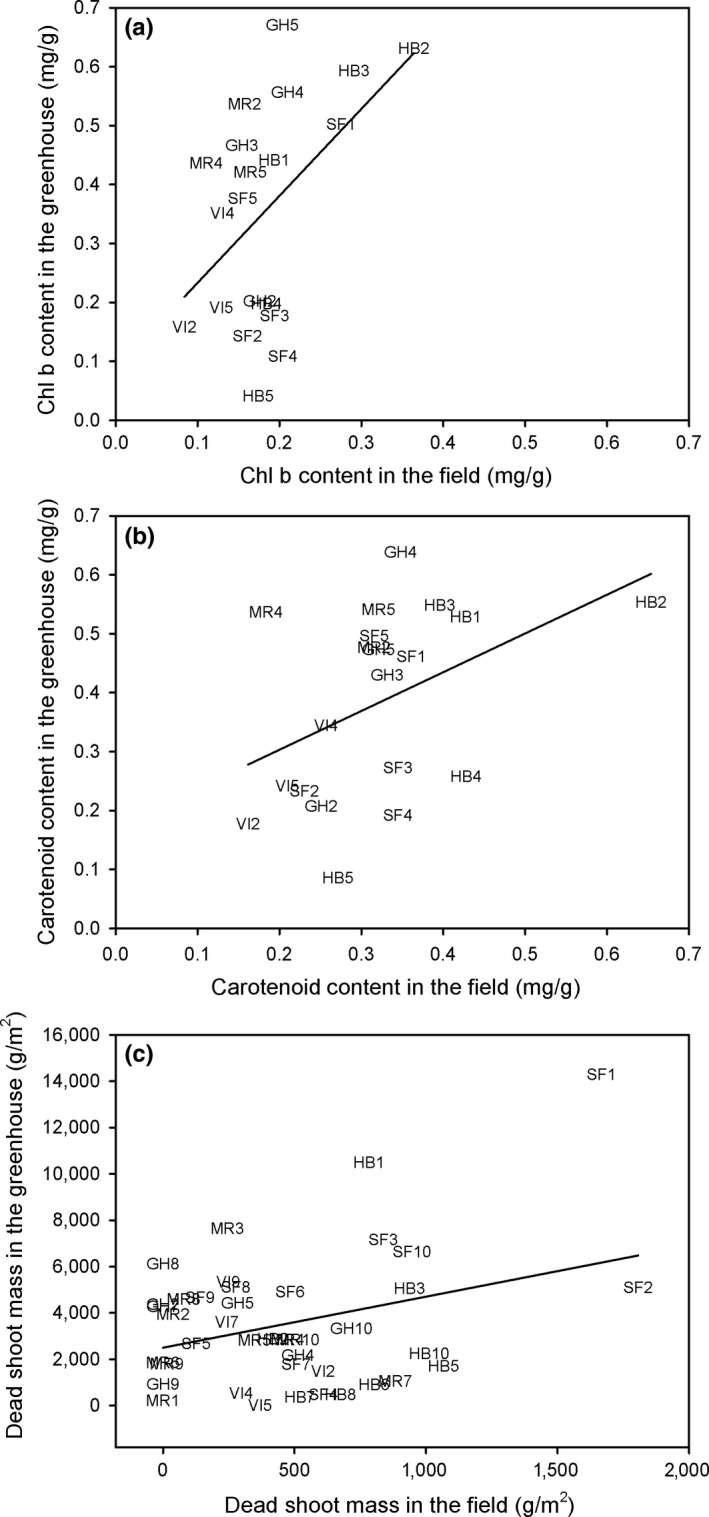
Relationships between foliar content of chlorophyll b (a) and carotenoids (b) and dead shoot mass (c) in the field and in a common‐garden experiment at individual level from five populations of *Spartina densiflora* along the Pacific coast of North America. Labels—Populations: VI, Vancouver Island; GH, Grays Harbor Estuary; MR, Mad River Estuary; HB, Humboldt Bay Estuary; SF, San Francisco Bay Estuary. Regression equation: (a) *y* = 0.09 + 1.48*x*; (b) *y* = 0.17 + 0.66*x*; (c) *y* = 2499.72 + 2.20*x*

Those individuals with higher values in the field also had higher values in the common‐garden experiment for contents of Chl b and Car and dead shoot mass (Chl b: *r* = .493, *n* = 20, *p* < .05; Car: *r* = .435, *n* = 20, *p* = .055; dead shoot mass: *r* = .328, *n* = 50, *p* < .05) (Figure 6). Moreover, those individuals with higher contents of Chl b also had higher contents of Car both in the field and in the glasshouse (field: *r* = .876, *n* = 25, *p* < .0001; glasshouse: *r* = .910, *n* = 25, *p* < .0001; Figure 6a,b).

## 4 DISCUSSION

Our results show that the populations of *Spartina densiflora* gradually invading the Pacific coast of North America along 12° of latitude from the late nineteenth century when it was first introduced to Humboldt Bay, until recently with the discovery of a new invasion in 2005 at Vancouver Island, are genetically very similar according to the chloroplast DNA sequences and the nuclear microsatellite loci investigated. This low genetic variation may be accentuated through the founder effect (Brzyski, Taylor, & McLetchie, 2014) for recent invasive populations that are going through bottlenecks due to genetic drift (Bilton, Paula, & Bishop, 2002; Dlugosch & Parker, 2008), which reduces diversity even in populations with high sexual reproduction rates (Kittelson & Boyd, 1997) promoting genetic recombination (Hartfield, 2016). Our results agree with those of Ayres et al. (2008) who recorded low genetic diversity among *S. densiflora* plants in San Francisco Bay and Humboldt Bay, which is consistent with the origin of San Francisco Bay population as transplants introduced from Humboldt Bay (Faber, 2000). Low genetic diversity is also encountered in *S. versicolor* populations from Europe which appear to be introduced plants from the Native American range of *S. patens* (Baumel et al., 2016). Our value of genetic differentiation among populations (Φ_PT_ = 0.159) is in the range of those reported previously for other invasive species such as *S. alterniflora* in China (*F*_ST_ = 0.234, Guo et al., 2015; *F*_ST_ = 0.100, Bernik, Li, & Blum, 2016), the polyploid red algae *Asparagopsis taxiformis* (Delile) Trevis. in the Mediterranean (Φ_PT_ = 0.249, being lower than in its native range, Andreakis et al., 2009), *Ambrosia artemisiifolia* L. in France (*F*_ST_ = 0.054, Genton, Shykoff, & Giraud, 2005), *Solidago canadensis* L. in Europe (*G*_ST_ (analogous to *F*_ST_) = 0.109, Moran et al., 2017), or *Mikania micrantha* Kunth in China (*F*_ST_ = 0.044, Geng, Chen, Cai, Cao, & Ou‐Yang, 2016).

These genetically similar *S. densiflora* populations showed high levels of phenotypic variability when growing in dissimilar environments in the field. Most of the interpopulation differences in plant traits recorded in the field disappeared when growing in common‐garden conditions, indicating that the observed variation in natural populations was the result of phenotypic plasticity (Castillo et al., 2014, 2016; Grewell et al., 2016). Nevertheless, the mean interpopulation phenotypic variability recorded for every plant trait in the common‐garden experiment was 37% (in comparison with the 56% recorded in the field), which pointed to another process, beyond phenotypic plasticity, that was also responsible for a significant amount of the recorded interpopulation phenotypic variation. In this sense, our study revealed that invasive *S. densiflora* populations had a similar, fixed, and high intrapopulation phenotypic variability for many plant traits in the field and in the common‐garden experiment (where it was higher than phenotypic plasticity for most of the recorded traits). Moreover, this intrapopulation phenotypic variability was especially high, irrespective of environmental conditions, for those traits showing also high phenotypic plasticity, suggesting that intrapopulation phenotypic variability in *S. densiflora* would be regulated through the same mechanisms as those regulating phenotypic plasticity. Ensuring high levels of intrapopulation phenotypic variability for key plant functional traits would allow *S. densiflora* to respond adequately to the changing environment (Zhao et al., 2016) of salt marshes, where local temporal and spatial changes on salinity, soil moisture, oxygenation, and flooding period are frequent (Adam, 1990; Castillo et al., 2000). The presence of intrapopulation phenotypic variability within species can result in faster range expansions than if only a single phenotype is present in the landscape (Elliott & Cornell, 2012).

Many of the studied plant traits had PI_*v*_ values higher than 50% in the field, and the mean PI_*v*_ for every trait was 54 ± 4%, within the range recorded for other invasive plant species (Funk, 2008) and native species along wide latitudinal ranges (Chun‐Can et al., 2014; Molina‐Montenegro & Naya, 2012; Zhao et al., 2012). Our results are also consistent with previous studies on other polyploid *Spartina* species (Castillo, Redondo, Wharmby, Luque, & Figueroa, 2005; Elsey‐Quirk et al., 2011; Thompson et al., 1991; Trnka & Zedler, 2000). *S. densiflora* is a heptaploid species of hybrid origin (Fortune et al., 2008), which most likely has played a key role in high phenotypic variability observed. Even in genetically depauperate populations (i.e., at the interindividual level), the intragenomic diversity (within individuals) resulting from both genome merger (hybridization) and genome duplication (polyploidy) increases the potential heterozygosity. At the examined loci, two alleles were most frequently encountered in the detected heterozygous genotypes (Table 2); more than two alleles were observed at the MS15 locus (the most common heterozygous genotype exhibiting three alleles) and the MS18 locus (heterozygous with 2 or 3 alleles). This is consistent with the hybrid (alloheptaploid) origin of this species (2*n* = 70, Ayres et al., 2008), which is believed derived from a tetraploid and a hexaploid ancestor in its native South American range (Ainouche et al., 2009; Fortune et al., 2008), although more alleles per locus could be expected in a heptaploid genotype. Genes duplicated by polyploidy (homeologs) can exhibit various gene expression evolution patterns which include alternative homoeologous gene silencing, subfunctionalization, neofunctionalization, and nonadditive parental gene expression such as expression of dominance or transgressive expression (Grover et al., 2012). Polyploidy has been repeatedly linked with plant invasion success; in particular, allopolyploidy (hybridization followed by genome multiplication) may lead to substantial epigenetic variation, allowing the expression of novel phenotypes with increased fitness and conferring genetic advantages linked to genetic diversity and gene expression (te Beest et al., 2011; Jackson, 2017). Ecotypic differentiation of *S. densiflora* has been described for populations in its native range related to environmental changes driven by latitude (Álvarez et al., 2009).

These genome dynamics likely support an increased range of potentially adaptive phenotypic variation and the ability of the species to invade diverse habitats. Our research suggests that the alloheptaploid nature of the *S. densiflora* genome (Ayres et al., 2008; Fortune et al., 2008) contributes to the responses of this invasive plant species to the changing environments in which it has colonized along the Pacific coast of North America. Phenotypic plasticity has been associated to increased competitive ability and fitness in many taxa, especially when coping with accelerated changing conditions (Hoffman & Sgro, 2011). The above‐reported high phenotypic variability would contribute to an overall “jack‐of‐all‐trades” result with less variability across environments in terms of population‐level consequences (Richards, Bossdorf, Muth, Gurevitch, & Pigliucci, 2006). Consequently, *S. densiflora* would have developed an adaptive phenotypic plasticity that can itself evolve (Crispo, 2008). *S. densiflora* occupies a wide variety of habitats from 23°20′S to 51°33′S latitude in its native range (Bortolus, 2006) and it has invaded very different estuarine habitats in the Iberian Peninsula (Nieva, Diaz‐Espejo, Castellanos, & Figueroa, 2001), where a variety of phenotypes are recognized (Castillo, Mateos‐Naranjo, Nieva, & Figueroa, 2008; Castillo, Rubio‐Casal, Luque, Figueroa, & Nieva, 2003; Nieva, Castellanos, Castillo, & Figueroa, 2005).

Understanding which traits of an invasive species show higher phenotypic variability may inform us about aspects of its aggressive behavior, which can be important to make predictions about its future spread using invasion models (Martina & von Ende, 2012). The plant traits exhibiting high (>60%) population phenotypic variability were leaf elongation and rolling and those related to aboveground architecture of tussocks, which have been reported as significant factors contributing to fitness (Cornelissen, Song, Yu, & Dong, 2014; Heckathorn & DeLucas, 1991; Kadioglu & Terzi, 2007; Schierenbeck, Fleitas, Miralles, & Simon, 2016). *Spartina densiflora* has invaded salt marshes in the southwest Iberian Peninsula, where aboveground architecture of its tussocks also varies among different habitats (Nieva et al., 2001). On the other hand, *S. densiflora* also occupies a wide variety of habitats in its native range along the South Atlantic coast of South America, where a high degree of phenotypic plasticity has been observed in its root system (Daleo & Iribarne, 2009), which was similar to the interpopulation root system variability observed in our study (ca. 60%). Grewell et al. (2016) studied the response to salinity of the same *S. densiflora* populations as those analyzed in this study. They found lower phenotypic plasticity values for *S. densiflora* in response to just salinity than what we found in the field where a mix of environmental factors such as sediment oxygenation, flooding period, latitude‐related abiotic factors, and biotic interactions were interacting. This comparison points to additive or even synergic effects, resulting from the combination of environmental factors on phenotypic plasticity.

Within‐population variation represented 84% of the total genetic variation coinciding with certain individual plants keeping consistent responses for three studied plant traits at different environmental conditions (in the field and in common‐garden conditions). This result is in agreement with the slight but significant increase in genetic differentiation when increasing geographic distance. Thus, those individuals from lower latitudes showing higher values for Chl *b* and Car contents and dead shoot biomass in the field also showed the higher values after more than 2 years in common‐garden conditions. Castillo et al. (2014) recorded higher photosynthetic pigment concentrations at lower than at higher latitudes in the same *S. densiflora* populations. When plants were grown in common‐garden conditions, these differences disappeared at the population level. However, our results show that they were kept at the individual level with some individuals from low latitudes (specially from Humboldt Bay) accumulating more Chl *b* and Car in the field and in the glasshouse and some individuals from high latitudes (specially from Vancouver Island) showing the opposite response. Thus, although environmental conditions, hybridization, and polyploidy largely determined plant traits in *S. densiflora*, our results point to a genetic or epigenetic component influencing certain traits due to changing selection pressures along a wide latitudinal range (Zhao et al., 2012). Ayres et al. (2008) recorded low detectable genetic variation in *S. densiflora* samples from San Francisco Bay, but ecotypic differentiation related to environmental changes driven by latitude and elevation along the tidal gradient has been described for *S. densiflora* populations in its native range (Álvarez et al., 2009; Di Bella et al., 2014). These results support the hypothesis that the fitness and range potential of invasive species can increase as a result of genetic or epigenetic divergence of populations along broad climatic gradients (Konarzewski, Murray, & Godfree, 2012).

Halophytes need to have efficient medium‐ and long‐distance dispersal mechanisms as they inhabit in salt marshes that are ephemeral ecosystems (Fagherazzi, 2013) due to relatively rapid succession development (Figueroa et al., 2003) and frequent and intense perturbations such as erosion (Castillo, Rubio‐Casal, Luque, Nieva, & Figueroa, 2002). The dispersion of many halophytes such as *S. densiflora* is carried out through hydrochorous propagules rafting on local and regional currents (Morgan & Sytsma, 2013), avoiding habitat fragmentation that may limit species range shift (Davis & Shaw, 2001). *S. densiflora* populations produce many viable seeds (‘Kittleson & Boyd, 1997; Nieva, Castellanos et al., 2001; Nieva, Diaz‐Espejo et al., 2001) and also colonize surrounding sediments by vegetative growth via tillering, producing dense tussocks through “phalanx” clonal growth (Nieva et al., 2005). In this content, the high levels of phenotypic plasticity and intrapopulation phenotypic variability of *S. densiflora* for key plant traits would promote seedlings to acclimate and to colonize new salt marshes with contrasted environmental conditions. *S. densiflora* started its invasion of the Pacific coast of North America in the late nineteenth century in Humboldt Bay (Spicher & Josselyn, 1985), being transplanted from there to San Francisco Bay in the late 1970s (Faber, 2000) and then expanding naturally its distribution northward to Grays Harbor in 2001 (Pfauth et al., 2007) and to Vancouver Island in 2005 (Morgan & Sytsma, 2010). In view of our results, multiple introductions are unknown but possible as the population from Vancouver Island was the most recent and one of the most genetically diverse. *S. densiflora* appears as a species that would not be very affected itself by climate change and sea‐level rise as it can disperse, establish, and acclimate to contrasted environments along wide latitudinal ranges. Global climate change may even favor some polyploid taxa that can tolerate stressful environments due to greater phenotypic plasticity (Meyerson et al., 2016).

In summary, our results show that *S. densiflora* that has invaded a broad latitudinal gradient along the Pacific coast of North America is characterized by low genetic divergence between populations, high phenotypic plasticity related to local conditions, and high and fixed intrapopulation polymorphism in some functional traits, which appears to be a key feature of this allopolyploid invasive species.

## CONFLICT OF INTEREST

None declared.

## AUTHOR CONTRIBUTIONS

J.M.C. and B.G.T. involved in the conceptualization, in the methodology, and in the investigation and wrote the original manuscript. E.F. involved in the conceptualization. B.J.G. involved in the conceptualization, in the methodology, and in the investigation and reviewed and edited the manuscript. D.V., H.R., J.K., O.L., S.D., and A.S. involved in the methodology. M.A. involved in the conceptualization, in the methodology, and in the investigation and reviewed and edited the manuscript.
